# Similar Patient Satisfaction and Quality of Life Improvement Achieved with TKA and THA According to the Goodman Scale: A Comparative Study

**DOI:** 10.3390/jcm12186096

**Published:** 2023-09-21

**Authors:** Maximiliano Barahona, Felipe Bustos, Tomás Navarro, Pablo Chamorro, Macarena Alejandra Barahona, Sebastián Carvajal, Julian Brañes, Jaime Hinzpeter, Cristian Barrientos, Carlos Infante

**Affiliations:** 1 Department of Orthopaedic Surgery, Hospital Clínico Universidad de Chile, Independencia, Santiago 8380420, Chilecainfantec@gmail.com (C.I.); 2 Department of Orthopaedic Surgery, Hospital del Salvador, Providencia, Santiago 7500922, Chile

**Keywords:** osteoarthritis, total knee arthroplasty, total hip arthroplasty, satisfaction, quality of life

## Abstract

**Background:** Total knee arthroplasty (TKA) and total hip arthroplasty (THA) are effective treatments for severe knee and hip osteoarthritis. Historically, TKA has been associated with lower satisfaction than THA, but recent advances in knee surgery have led to lower dissatisfaction rates. This study aimed to compare the satisfaction and self-reported improvement in the quality of life of two cohorts of patients who underwent TKA and THA, respectively. **Methods:** This observational study compared two previously published cohorts of patients who underwent THA and TKA in a single university center. The Goodman scale was used to assess satisfaction and self-perception of improved quality of life after TKA and THA at a minimum one-year follow-up. Propensity score matching was used to balance age, gender, and follow-up between groups. Significance was set at 0.05. **Results:** The study included a total of 105 THAs and 131 TKAs. Both groups had high levels of satisfaction with pain relief, ability to do house/yard work, and overall satisfaction, with above 90% satisfaction rates. Regarding improvement in quality of life, both groups had 86% of patients reporting improvement as “much better.” After propensity score matching, no significant difference was found between THA and TKA for any of the comparisons made using the Goodman scale. **Conclusions:** The study showed that both TKA and THA resulted in high levels of satisfaction and improvement in quality of life. There was no significant difference in satisfaction rates between TKA and THA, contrary to the historical trend of lower satisfaction rates for TKA.

## 1. Introduction

Osteoarthritis is pathologically characterized by the localized loss of cartilage, remodeling of adjacent bone, and associated inflammation that may lead to severe joint damage, pain, and deterioration in quality of life. The hip and knee are among the joints most commonly affected by this disease [1].

Total knee arthroplasty (TKA) and total hip arthroplasty (THA) are effective treatments for severe knee and hip osteoarthritis. However, historically, TKA has been associated with lower satisfaction than THA [2]. THA has been recognized as the superior surgery over the last century due to its positive impact on quality of life [3], while TKA has not generated a similar level of impact. Nevertheless, significant advances have been made in knee surgery over the last two decades, such as improvements in pain management, implant technology, greater dissemination of the surgery, and even the incorporation of navigation and robotic assistance [4]. Despite the classic post-TKA dissatisfaction threshold being set at 20%, a recent meta-analysis suggests that the current dissatisfaction rate is around 10% [5].

Goodman et al. [6] recently published a patient-reported scale that assesses satisfaction and self-perception of improved quality of life after TKA and THA, which was validated in Spanish [7]. Having an instrument that measures patient satisfaction for both procedures facilitates the comparison between these two procedures [8].

The aim of this study is to compare two cohorts of patients, one who underwent TKA and the other THA, in the same university hospital. The null hypothesis is that both cohorts will present similar percentages of satisfaction with a minimum of one-year follow-up.

## 2. Methods

This study is an observational design that compares two previously published cohorts of patients who underwent THA and TKA in the same university hospital. The THA cohort included patients who underwent conventional uncemented primary hip replacement for hip osteoarthritis between 2018 and 2019 [7], while the TKA cohort included patients who underwent conventional cemented total knee replacement without patella resurfacing using an anterior stabilized insert (CRAS) between 2018 and 2020 [9]. This study was approved by our local ethics committee.

The THA cohort was initially gathered to validate the Spanish version of the Goodman scale. These patients were assessed telephonically using this validated scale in 2020. In contrast, the TKA cohort was engaged to evaluate patient-reported outcomes, encompassing satisfaction. These patients were assessed in-person in 2022 by a sole evaluator utilizing the validated Spanish Goodman scale. From the available 126 THA candidates for evaluation, 105 (83.4%) were successfully reached and consented to be evaluated for study participation [7]. Similarly, of the 163 TKA candidates available, 131 (80.4%) were successfully contacted and consented to evaluation in the primary study [9]. Demographic data, including age at the time of surgery, gender, and follow-up durations, were recorded for both cohorts.

The Goodman scale comprises two sections designed to assess satisfaction and self-perceived improvement in quality of life [6,7,10]. Section A assesses satisfaction using four questions, each with five possible answers (ranging from “very unsatisfied” (0 points) to “very satisfied” (4 points)). These four questions can be summarized by calculating the average score and multiplying by 25, with the minimum score of 0 being “very dissatisfied” and the maximum score of 100 being “very satisfied.” A score of at least 75 was considered satisfactory as it represents an average of 3 on the four questions. A score of 75 or above was considered “satisfied,” as this reflects an average score of 3 across the four questions. In addition, each question in this section was dichotomized as “satisfied” if the response was 3 or 4 and “unsatisfied” if the answer was 2 or lower.

Section B is a single question that inquires about the patient’s perceived improvement in quality of life after surgery, offering six possible answers, from “more than I ever dreamed” (6 points) to “worse” (0 points). For analytical purposes, this question was categorized into “much better” if the response was 5 or 6 and categorized into “same or worse” if the answer was 4 or less.

Continuous variables were summarized using the median and range, while categorical variables were summarized using frequency and percentage. The Wilcoxon rank-test (a median comparison of unpaired independent samples) was used to compare continuous variables, and Fisher’s exact test was used to compare categorical variables. A propensity logistic matched score was estimated using three matches per observation to account for the variability in age at surgery, gender proportion, and follow-up. A coefficient was considered significant if the probability achieved was less than 0.05. Stata v17 (StataCorp, College Station, TX, USA) was used for all analyses.

## 3. Results

The study included a total of 105 THA and 131 TKA patients. While the median age was similar in both groups, there was a significant difference in the gender distribution and the follow-up period, with THA having a higher proportion of female patients and a shorter follow-up time (Table 1).

Overall, both THA and TKA groups had high levels of satisfaction with pain relief, ability to do house/yard work, and overall satisfaction, with above 90% satisfaction rates (Table 2). Satisfaction with recreational activities was also high but below 90%; the THA cohort reached 89.5% (*n* = 94) and the TKA cohort reached 84.7% (*n* = 111), with no significant difference. Consistently, no significant difference was found using 75 points as the threshold for Section A (Table 2). Regarding improvement in quality of life, both groups had 86% of patients reporting improvement as “much better”. (Table 2).

After propensity score matching, no significant difference was found between THA and TKA for any of the comparisons made using the Goodman scale (Table 3). The standardized differences were below one in all three independent variables: age, follow-up, and gender (Table 4).

## 4. Discussion

This single-center cohort study compared TKA and THA satisfaction and quality of life improvement using the Goodman scale. The results showed that both procedures had high levels of satisfaction in categories such as pain relief, ability to do house/yard work, overall satisfaction, and improvement in quality of life. The satisfaction rate for recreational activities was also high but slightly lower than other categories, with no significant difference between the two cohorts.

The results of this study contradict the historical reports from the first decade of the current century that suggested that TKA had lower satisfaction rates than THA [11,12], which can be attributed to significant advances in knee surgery over the last decade, such as improvements in pain management [13,14], implant technology and design [15], and even the incorporation of navigation and robotic assistance [16].

Recent literature has reported similar rates of good results using patient-reported outcomes for both procedures, including the Australian registry, where good results were reported in 90.3% of TKA cases (*n* = 311) and 89.5% of THA cases (*n* = 516) [17]. Recent meta-analyses found that unsatisfaction rates were 10% for TKA [5] and 7% for THA [18]. Halawi et al. [19] reported that persistent pain was the most common cause for dissatisfaction in both surgeries and that patients also consider the care delivery process in their satisfaction assessment.

A significant strength of the present study is the consistent use of the Goodman scale across both cohorts, bolstering the conclusions drawn from the aforementioned studies. Since its introduction, the Goodman scale has showcased a strong correlation with other patient-reported measures. The original Goodman study reported a significant moderate correlation with both HOOS and KOSS [6]. In its Spanish validation, a similar correlation was noted with the OHS [7]. Moreover, in knee patients, the KUJALA, KOOS quality of life, and WOMAC scales can predict the Goodman score, yielding an area under the ROC curve of 0.82 (95% CI: 0.70 to 0.94) [9]. Furthermore, Ulivi et al. [10] identified a moderate-to-strong correlation between the Goodman score and both EQ-5D and SF-12. It is imperative to highlight that Ulivi et al. [10] also observed a 30% ceiling effect when using the Goodman score. However, this ceiling effect is consistent across both TKA and THA groups, which could introduce bias but would do so for both cohorts equally. This suggests that, despite the potential confounding factor of comparing THA and TKA using a single scale, evidence supports the Goodman scale’s consistent correlation with other more established tools.

The Goodman scale, as employed in this study, stands out as a pivotal instrument in gauging patient satisfaction across both procedures, thus facilitating insightful comparisons between them [8]. To enhance the validity of the results in this observational study, the researchers employed a propensity score matching method to account for the unbalanced follow-up time and gender ratio [20].

In addition, although the cohort of patients with THA had a shorter follow-up period in this study, Schmitz et al.’s indicated that satisfaction with this surgery does not diminish with longer follow-ups [21]. While female patients have been reported to be at a higher risk of developing chronic pain and dissatisfaction after TKA [22], our previous study and the meta-analysis conducted by DeFrance et al. showed no difference in satisfaction rates by gender [5,9]. Furthermore, the development of chronic pain has been associated with managing perioperative pain, sleep disorders, personality traits, and psychiatric disorders such as anxiety rather than being solely linked to gender [23,24,25].

Given that both procedures have similar satisfaction rates, public policies in each country should aim to favor access to both procedures. The OECD uses the number of arthroplasties performed per 100,000 inhabitants as a health indicator [26]. For example, in Chile, only access to THA is guaranteed by law [27], leaving the population requiring TKA relegated to significantly prolonged waiting lists [28].

The primary limitation of this study arises from comparing two cohorts where the Goodman scale was applied for purposes that were different from the current study’s focus, even though it was the main outcome in both original studies. Like many observational studies, there were imbalances between the groups regarding the proportion of females and follow-up duration. However, employing the propensity score analysis effectively attenuates this bias. Importantly, we did not perform analysis with factors such as diabetes, history of chronic pain, and psychiatric disease history. This omission prevents us from adjusting for these significant variables that undoubtedly influence patient satisfaction [25,29,30]. Additionally, while the baseline physical activity level was not recorded and does affect the outcome of the arthroplasty [31,32], the Goodman scale’s design—which specifically inquiries about patients’ satisfaction relative to their pre-surgical baseline—helps in counteracting this limitation. Notably, BMI data were absent for the THA group, barring its inclusion in the analysis. Patients with a higher BMI have an increased risk of complications [33]; however, it is worth noting that despite these potential complications, significant improvements in functional outcomes have been seen with elevated BMI [34,35]. Remarkably, within the TKA group, it has been reported that a higher BMI was correlated with enhanced satisfaction compared to their baseline [9].

Other limitations of this study include being a single-center study and having a limited sample size. While our facility might be considered low volume on an international scale, it boasted the highest number of TKA procedures between 2004 and 2019 and ranked in the top ten for THA in our nation [36]. Between 2017 and 2020, the THA/TKA ratio was nearly one, slightly favoring THA. However, post-COVID-19, this ratio shifted towards TKA in our institution. As such, our university center possesses considerable expertise in both surgical procedures.

## 5. Conclusions

The study showed that both TKA and THA resulted in high levels of satisfaction and improvement in quality of life. There was no significant difference in satisfaction rates between TKA and THA, contrary to the historical trend of lower satisfaction rates for TKA.

## Figures and Tables

**Table 1:** Comparison of sample size, age at surgery, gender, and follow-up between the cohorts.

	TKA	THA	Test
Sample size	131	105	
Age	66 (47 to 88)	65 (22 to 85)	*p* = 0.47 (w)
Female	73 (55.8%)	76 (72.38%)	*p* = 0.01 (f)
Follow up	2.7 (1 to 5)	1 (1 to 2)	*p* < 0.00 (w)

**Table 2:** Comparison of satisfaction and improvement after THA and TKA.

Item	TKA	THA	Test
Score Goodman A	100 ((0 to 100) 88 to 100)	100 ((0 to 100) 88 to 100)	0.23 (w)
>75 points Goodman A	119 (90.8%)	96 (91.4%)	0.99 (f)
Satisfaction w/Pain relief	123 (93.9%)	97 (92.3%)	0.80 (f)
Satisfaction w/Ability to do house/yard work	121 (92.4%)	97 (92.4%)	0.99 (f)
Satisfaction w/Ability to do recreational activities	111 (84.7%)	94 (89.5%)	0.33 (f)
Overall Satisfaction	121 (92.4%)	98 (93.3%)	0.81 (f)
Improvement “Much Better”	113 (86.3%)	91 (86.7%)	0.99 (f)

Abbreviations: w = with.

**Table 3:** Comparison after propensity matching analysis for age, gender, and follow-up between TKA and THA cohorts.

Item	THA vs. TKA	*p*
Score Goodman A	−0.61 (−4.12 to 2.90)	0.734
>75 points Goodman A	0.01 (−0.04 to 0.05)	0.845
Satisfaction w/Pain relief	0.01 (−0.03 to 0.05)	0.666
Satisfaction w/Ability to do house/yard work	0 (−0.04 to 0.04)	0.99
Satisfaction w/Ability to do recreational activities	−0.03 (−0.09 to 0.02)	0.205
Overall Satisfaction	−0.01 (−0.04 to 0.04)	0.833
Improvement “Much Better”	0.03 (−0.06 to 0.12)	0.507

Abbreviations: w/ = with; vs. = versus.

**Table 4:** Summarized standardized differences in the raw and matched data.

Variable	Raw	Matched
Age at surgery	−0.25	0.26
Years F.U.	−1.77	−0.65
Gender	−0.35	0.55

Abbreviations: F.U. = follow-up.

## Data Availability

Data are available on reasonable request to the corresponding author, who must previously consult the institution’s ethics committee.
